# A thermodynamic assay to test pharmacological chaperones for Fabry disease^☆^

**DOI:** 10.1016/j.bbagen.2013.12.018

**Published:** 2014-03

**Authors:** Giuseppina Andreotti, Valentina Citro, Antonella Correra, Maria Vittoria Cubellis

**Affiliations:** a Istituto di Chimica Biomolecolare, CNR, Pozzuoli, Italy; b Istituto di Genetica e Biofisica ‘A. Buzzati Traverso,’ CNR, Napoli, Italy; c Dipartimento di Biologia, Università Federico II, Napoli, Italy; d Istituto di Biostrutture e Bioimmagini, CNR, Napoli, Italy

**Keywords:** PC, pharmacological chaperones, FD, Fabry disease, AGAL, lysosomal alpha-galactosidase, DGJ, 1-deoxy-galactonojirimycin, CD, circular dichroism, Pharmacological chaperone, Lysosomal storage disorder, Urea-induced unfolding, Limited proteolysis, Cell lysate

## Abstract

**Background:**

The majority of the disease-causing mutations affect protein stability, but not functional sites and are amenable, in principle, to be treated with pharmacological chaperones. These drugs enhance the thermodynamic stability of their targets. Fabry disease, a disorder caused by mutations in the gene encoding lysosomal alpha-galactosidase, represents an excellent model system to develop experimental protocols to test the efficiency of such drugs.

**Methods:**

The stability of lysosomal alpha-galactosidase under different conditions was studied by urea-induced unfolding followed by limited proteolysis and Western blotting.

**Results:**

We measured the concentration of urea needed to obtain half-maximal unfolding because this parameter represents an objective indicator of protein stability.

**Conclusions:**

Urea-induced unfolding is a versatile technique that can be adapted to cell extracts containing tiny amounts of wild-type or mutant proteins. It allows testing of protein stability as a function of pH, in the presence or in the absence of drugs. Results are not influenced by the method used to express the protein in transfected cells.

**General significance:**

Scarce and dispersed populations pose a problem for the clinical trial of drugs for rare diseases. This is particularly true for pharmacological chaperones that must be tested on each mutation associated with a given disease. Diverse in vitro tests are needed. We used a method based on chemically induced unfolding as a tool to assess whether a particular Fabry mutation is responsive to pharmacological chaperones, but, by no means is our protocol limited to this disease.

## Highlights

• Pharmacological chaperones stabilize the folded state of proteins.
• Only some Fabry mutations can be treated with pharmacological chaperones.
• Urea-induced unfolding represents a novel assay to test the efficiency of drugs.
• The test with urea can be applied to a tiny amount of mutants in raw extracts.
• Responsiveness of Fabry mutations to drugs can be tested with urea-induced unfolding.

## Graphical abstract

**Figure:**
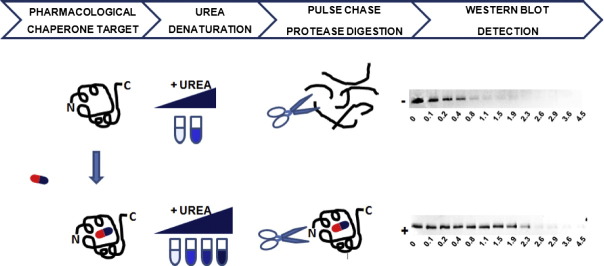

## 1 Introduction

The reduction in protein stability is the most common cause of monogenic diseases [1]. This knowledge paves the way to a new therapeutic approach. Pharmacological chaperones (PC) are small molecules that stabilize the mutant proteins, increase their intracellular concentration and consequently their intracellular activity. Because of their effect on the apparent stability of proteins they can also be described as “thermodynamic drugs”. The therapeutic approach with PC is currently being tested not only for lysosomal storage diseases [2] like Fabry [3], [4], Gaucher [5], [6] or Pompe [7], [8], but also for other diseases like Phenylketonuria [9].

In order to develop new methodologies to test PC, we used Fabry disease (FD) as a model system.

Fabry is an X-linked disease which affects not only male individuals, but also heterozygote female carriers, although in a milder form. It is due to mutations in the gene encoding lysosomal alpha-galactosidase (AGAL) [HGNC: GLA; UNIPROT: AGAL_HUMAN], a dimeric protein, synthesized and glycosylated in the endoplasmic reticulum and transported into lysosomes. The clinical picture of FD is rather complex because more than 400 missense mutations have been described and a good share of them is private. There is already an approved therapy for FD which consists in the infusion of the recombinant human protein (for a review on FD [10]). Unfortunately this therapy is very expensive and in many cases it causes the formation of antibodies against the exogenous protein [11]. For some mutations PC offer an alternative therapeutic approach. Chemicals to be used as PC can be found exploiting their ability to stabilize the wild type enzyme. Once they are found, they must be tested on each mutation because only a percentage of the cases will be responsive. This holds for FD and is in general true also for other diseases. An imino-sugar that resembles galactose, 1-deoxy-galactonojirimycin, also known as AT1001 or DGJ, is in clinical trial phase 3 for FD [12]. Some methods have been proposed [13], [14], [15], [16] to predict the cases which should be responsive to this drug, but tests in vitro are preferable. Classical methods to evaluate thermodynamic stability, which are very useful to find chemicals effective on wild type enzymes, are not feasible for the screening of mutants, because they require milligrams of purified proteins. Currently the effectiveness of PC is evaluated on patient's cells cultured in a medium supplemented or not with the drug [17], [18], [19], [20], [21]. Mutants are quantified by western blot or assayed enzymatically in the cell extracts without prior purification. Alternatively the half life of the mutants can be measured by pulse chase, but in these cases radioactive labeling is required [22]. Unfortunately patient derived xenografts or cells might not be available and in any case are not useful for female patients who are a mosaic of cells expressing either wild type or mutant AGAL. Transfected cells, which offer a useful surrogate to patient cells [18], [21], [22], [23], [24], [25], [26], [27], also present some disadvantages. In fact, under the high expression conditions that are usually employed, biosynthetic machinery might be saturated preventing the correct assessment of drug induced mutant stabilization. Moreover, results are reliable only if the efficiency of transfections in positive and negative controls, i.e. cells treated or not treated with PC, is exactly the same.

A good solution to test PC would be to adapt a classic method to measure thermodynamic stability of mutants produced in the cells in a small quantity and unpurified, in the presence or in the absence of the drug. In this article we propose to borrow a thermodynamic assay which is usually employed to assess protein stability for another purpose, that is testing drug responsiveness, and we suggest to use unfolding induced by urea [25]. Wild type AGAL was challenged with urea and results obtained monitoring unfolding with circular dichroism, pulse proteolysis or pulse proteolysis followed by western blot were compared. The last method which is designed for experiments on cell extracts containing low amounts of the protein of interest, was tested on four AGAL mutants. One of these, L300F-AGAL (c.898C>T, p.L300F) was produced using different transfection protocols in order to assess the reproducibility of results. Different pH buffers were used to mimic the conditions encountered by the protein in different cellular compartments.

## 2 Results

### 2.1 Chemically induced alpha-galactosidase unfolding

**Fig. 1:**
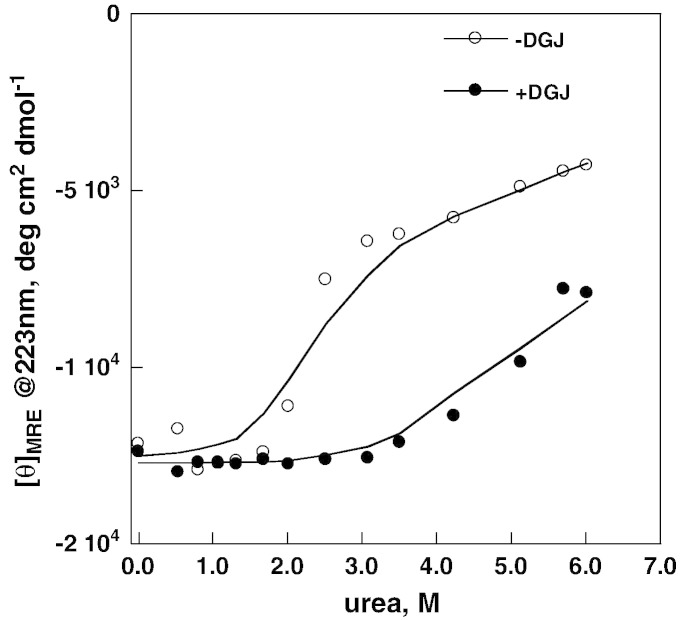
Urea-induced melting profile of wild-type lysosomal alpha-galactosidase (Fabrazyme®) recorded by circular dichroism. The protein (0.3 mg/ml in McIlvaine buffer at pH 7.4) was equilibrated with urea (from 0 to 6 M) in the presence of 1-deoxy-galactonojirimycin (DGJ) 40 μM or not, then ellipticity at 223 nm was recorded. Data were expressed as mean residue ellipticity.

AGAL (Fabrazyme®)(6 μM per monomer concentration) was incubated at 20 °C in McIlvaine buffer pH 7.4 in the presence of various concentrations of urea until unfolding equilibrium was obtained and a 16–18 h incubation was considered adequate to reach equilibrium in the subsequent experiments. We find a strong stabilizing effect of DGJ against urea denaturation, a sharp transition in the case of apo-enzyme and a broad transition in the case of the complexed enzyme (Fig. 1) Although thermally or chemically induced unfolding describes different processes, the reduced steepness of the denaturation curve in the presence of the drug confirms the results obtained by Petsko and coworkers [28] who found a coincidence of calorimetric and van't Hoff enthalpies for the free enzyme, but not for the complexed enzyme. A two state model can account for the unfolding of the apo-enzyme, but not for that of the complex. For this reason our analysis is limited to the midpoint urea concentration C_0.5_ of the normalized data as an operative measure of protein stability. This value can be determined quite accurately and is little affected by the mechanism of the unfolding. We are aware of the fact that a precise measure of the free energy of unfolding in water, which is outside the scope of this paper, would require the evaluation of the dependence of the free energy on denaturant concentration, i.e. the assessment of m-values [29]. However this value cannot be determined very accurately and its correlation with stability has been well documented mostly for monomeric proteins that unfold with a two-state mechanism and without ligands.

In the absence of DGJ C_0.5_ is at 2.0 ± 0.2 M urea (Fig. 1) whereas, due to the strong stabilizing effect of the drug and consequently to the difficulty of determining a true endpoint of the melting curve, in the presence of DGJ only an approximate C_0.5_ can be estimated above 4 M urea.

**Fig. 2:**
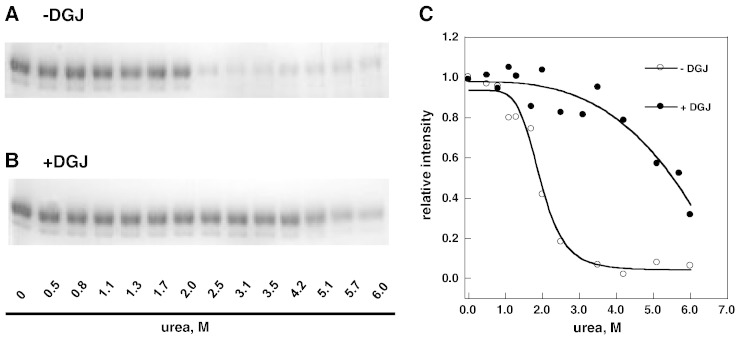
Urea-induced melting profile of wild-type lysosomal alpha-galactosidase (Fabrazyme®) recorded by pulse-proteolysis and SDS-PAGE analysis. The protein (0.3 mg/ml in McIlvaine buffer at pH 7.4) was equilibrated with urea (from 0 to 6 M) in the presence of 1-deoxy-galactonojirimycin (DGJ) 40 μM or not, then an aliquot of each sample was subjected to pulse proteolysis with thermolysin (1 min at 37 °C, 1:5 protease to substrate ratio) and then analyzed by SDS-PAGE. The protein was visualized by Coomassie Blue Staining (− DGJ, panel A; + DGJ, panel B). The intensity of the bands was quantified and expressed as relative intensity (panel C).

The same samples, which had been analyzed by circular dichroism (CD), were incubated with thermolysin (1:5 protease to Fabrazyme® ratio by weight). After having inactivated the protease by addition of EDTA, the samples were analyzed by SDS-PAGE (Fig. 2 panels A and B). The intensity of the bands was quantified and the profiles obtained (Fig. 2 panel C) confirm the stabilizing effect of DGJ. In particular we observe that the transition in the absence of DGJ is sharp whereas a more complex unfolding process occurs in the presence of DGJ. Hence the effect observed with a “classic” method, i.e. urea-induced denaturation monitored by circular dichroism (Fig. 1), is reproduced with pulse-proteolysis [30].

### 2.2 Scaling down chemically induced alpha-galactosidase unfolding for the analysis of mutants

**Fig. 3:**
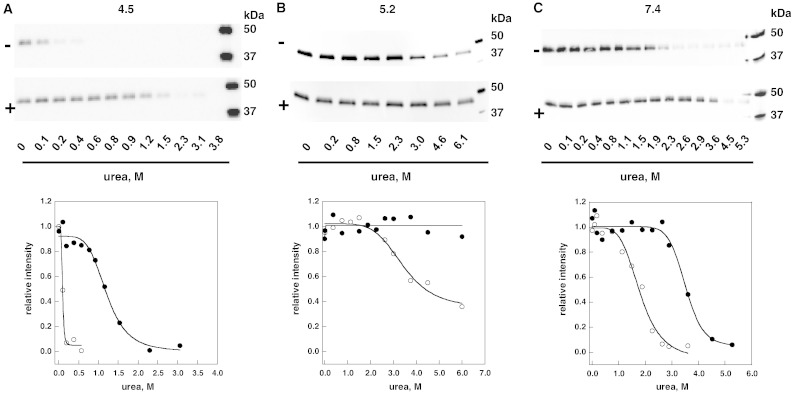
Urea-induced unfolding profiles of wild-type lysosomal alpha-galactosidase present in raw cell extracts recorded by pulse-proteolysis and western-blot. Lysates of COS7 cells expressing wild-type lysosomal alpha-galactosidase were mixed with the denaturant to obtain final urea concentrations ranging from 0 to 6 M. The experiment was conducted in McIlvaine buffer at pH 4.5 (panel A), 5.2 (panel B) or 7.4 (panel C) either in the absence or in the presence of 1-deoxy-galactonojirimycin (DGJ) 40 μM. Pulse proteolysis (1 min at 37 °C, 1:5 protease to substrate ratio) was performed after the equilibrium was reached to digest unfolded protein and then analyzed by western-blot. Thermolysin was used when operating at pH 5.2 or 7.4, pepsin when operating at pH 4.5. The intensity of the bands was quantified and data were expressed as fraction of the zero urea sample.

AGAL was produced in COS7 cells transiently transfected with an expression vector. Whole cell extracts were aliquoted and incubated with variable concentration of urea, in the presence or in the absence of 40 μM DGJ. After 16–18 h of equilibration, the unfolded proteins were digested proteolytically and the residual AGAL was quantified by western blot. Three sets of experiments were conducted at different pHs and are summarized in Fig. 3.

Thermolysin was added (1:5 ratio with total protein in the extracts by weight) when the denaturation had been carried out at pH 7.4 or 5.2. Pepsin was added (1:5 ratio with total protein in the extracts by weight) when the denaturation had been carried out at pH 4.5.

**Fig. 4:**
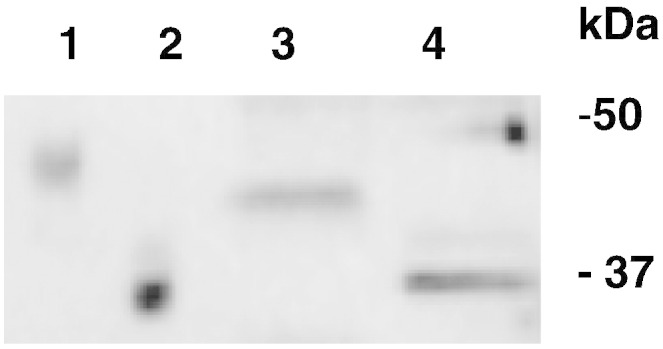
Comparison between glycosylation states of wild-type lysosomal alpha-galactosidase produced in COS7 cells and Fabrazyme®. Fabrazyme® and lysates of COS7 cells expressing wild-type lysosomal alpha-galactosidase were incubated overnight at 37 °C in the presence of N-Glycosidase F. The samples were pre-treated with SDS 0.1% and EDTA 20 mM, boiled and cooled and NP-40 was added to 0.7%. Samples were compared by SDS-PAGE and western blot: Fabrazyme® not treated (lane 1) or treated with N-Glycosidase F (lane 2); wild-type lysosomal alpha-galactosidase expressed in COS7 non-treated (lane 3) or treated with N-Glycosidase F (lane 4).

Fig. 3 panel C shows that at neutral pH DGJ stabilizes the protein shifting the midpoint concentration from 1.8 ± 0.1 M to 3.5 ± 0.2 M urea. C_0.5_ is slightly lower for the human enzyme expressed in COS7 cells than for Fabrazyme®. This can be explained by a different glycosylation of the enzyme. In Fig. 4 we compare AGAL produced in COS7 cells to Fabrazyme®. Their electrophoretic mobilities are different, but become the same upon treatment with N-Glycosidase F.

The enzyme is more stable at pH 5.2 (Fig. 3 panel B) compared to pH 7.4 (Fig. 3 panel C) with a C_0.5_ approximately at 4 M urea in the absence of the DGJ and it is resistant to denaturation even at 6 M urea in the presence of the drug. On the other hand, we observe a reduction in the stability at pH 4.5 and C_0.5_ values drop to 0.1 M in the absence of DGJ or to 1.2 M urea in the presence of DGJ.

**Fig. 5:**
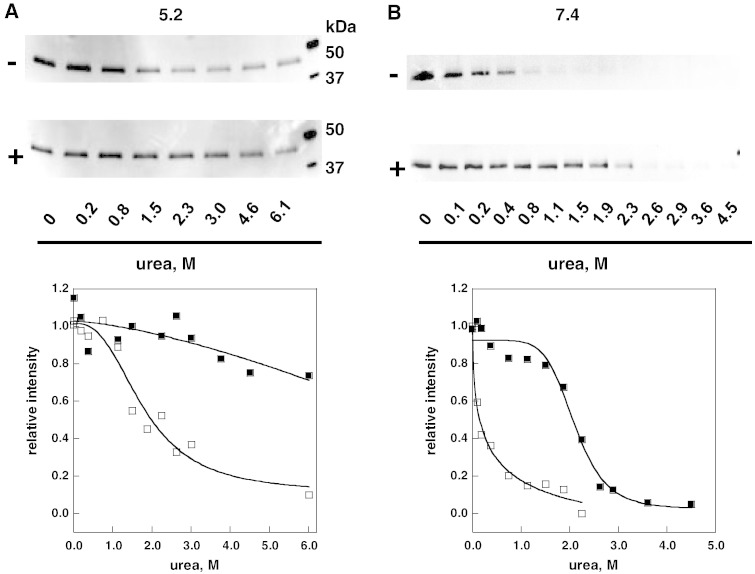
Urea-induced unfolding profiles of L300F lysosomal alpha-galactosidase present in raw extracts recorded by pulse-proteolysis and western-blot. Lysates of COS7 cells expressing L300F lysosomal alpha-galactosidase were mixed with the denaturant to obtain final urea concentrations ranging from 0 to 6 M. The experiment was conducted in McIlvaine buffer at 5.2 (panel A) or 7.4 (panel B) either in the absence or in the presence of DGJ 40 μM. Pulse proteolysis (1 min at 37 °C, 1:5 thermolysin to substrate ratio) was performed after the equilibrium was reached to digest unfolded protein and then analyzed by western-blot. The intensity of the bands was quantified and data were expressed as fraction of the zero urea sample.

L300F-AGAL [31] is a mutant with less than 10% of the wild type activity. COS7 cells expressing this mutant recover activity when they are exposed to DGJ [27]. We tested thermodynamic stability of L300F-AGAL by challenging it with urea. A single transfection in COS7 cells was required to produce the extract, 50 μg proteins in total, that was aliquoted and treated with different concentrations of urea in the presence or in the absence of DGJ. Fig. 5 shows the results obtained at pH 5.2 (panel A) or 7.4 (panel B).

This experiment demonstrates that the mutant is less stable than wild type both at neutral and at acidic pH, yet it becomes as stable as the wild type when treated with DGJ. Moreover, we observe that L300F-AGAL is more stable at pH 5.2 than at pH 7.4.

**Fig. 6:**
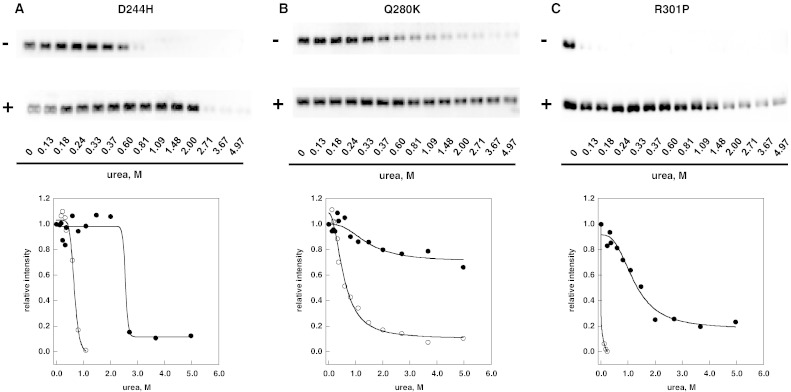
Urea-induced unfolding profiles of D244H, Q280K and R301P lysosomal alpha-galactosidase mutants present in raw cell extracts recorded by pulse-proteolysis and western-blot. Lysates of COS7 cells expressing D244H (panel A), Q280K (panel B) or R301P (panel C) lysosomal alpha-galactosidase were mixed with the denaturant to obtain final urea concentrations ranging from 0 to 5 M. The experiment was conducted in McIlvaine buffer at pH 7.4 either in the absence or in the presence of DGJ 40 μM. Pulse proteolysis (1 min at 37 °C, 1:5 thermolysin to substrate ratio) was performed after the equilibrium was reached to digest unfolded protein and then analyzed by western-blot. The intensity of the bands was quantified and data were expressed as fraction of the zero urea sample.

**Fig. 7:**
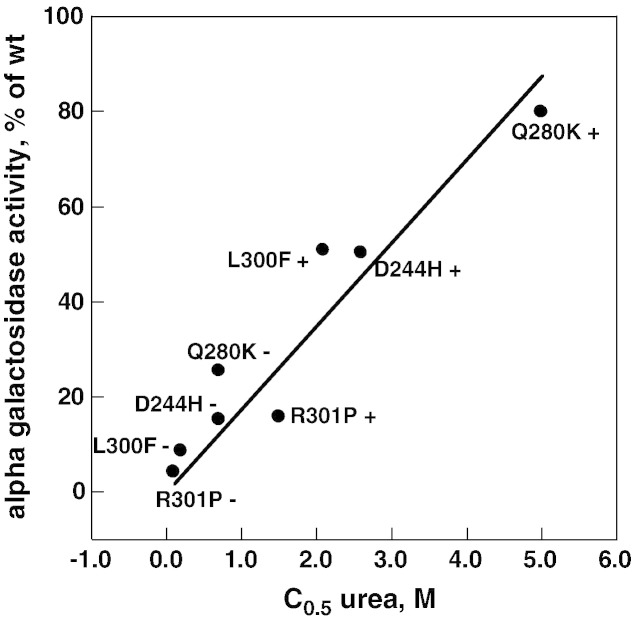
Correlation between urea C_0.5_ and alpha galactosidase increase. Urea concentration was from Fig. 5, Fig. 6 (this paper); alpha galactosidase activity is expressed as mut_activity + DGJ / WT_activity − DGJ × 100. The Pearson correlation coefficient is 0.94.

Besides L300F-AGAL, other mutations associated with classic clinical manifestations, Q280K-AGAL (c.838C>A, p.Q280K) [32], D244H-AGAL (c.730G>C, p.D244H) [33], R301P-AGAL (c.902G>C, p.R301P) [34] were tested at pH 7.4 with increasing amounts of urea, limited proteolysis and western blot. Results are shown in Fig. 6. We chose neutral conditions because they reflect the environment of the endoplasmic reticulum and the same expression protocol based on Lipofectamine® 2000 which had already been exploited for a previous experiment [27]. In that case transfected cells were cultured in a medium supplemented or not with the drug and AGAL activity was assayed enzymatically. C_0.5_ values derived by the plots in Fig. 5 panel B or Fig. 6 correlate with the AGAL activity measured in cell extracts [27] (Fig. 7 Pearson correlation coefficient r = 0.94).

**Fig. 8:**
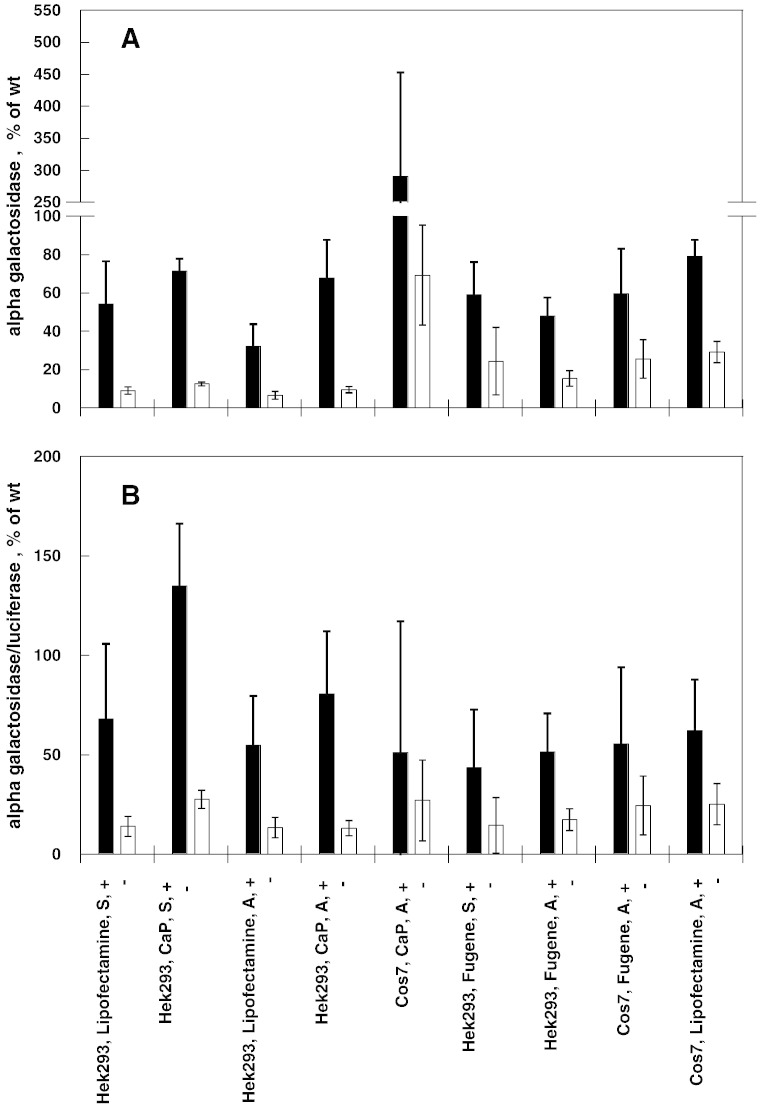
Alpha-galactosidase activity resulting from 1-deoxy-galactonojirimycin administration in transfected cells. Comparison between different cell hosts and transfection methods. COS7 or HEK293 cells were co-transfected with pCMV6-AC harboring L300F-AGAL gene and with pMIR vector harboring the luciferase reporter gene. Transfections were performed with Lipofectamine® ltx, Fugene® HD or CalPhos™ Mammalian Transfection Kit, either using cell in adhesion (A) or in suspension (S). Cells were than cultivated with (+) or without (−) 0.02 mM DGJ. After cell lysis AGAL activity and luciferase activity were measured. Four independent experiments were performed in the presence of DGJ, two without DGJ. Alpha galactosidase activity (expressed as mut_activity / WT_activity − DGJ × 100) was normalized by protein concentration in panel A and by luciferase activity in panel B.

**Fig. 9:**
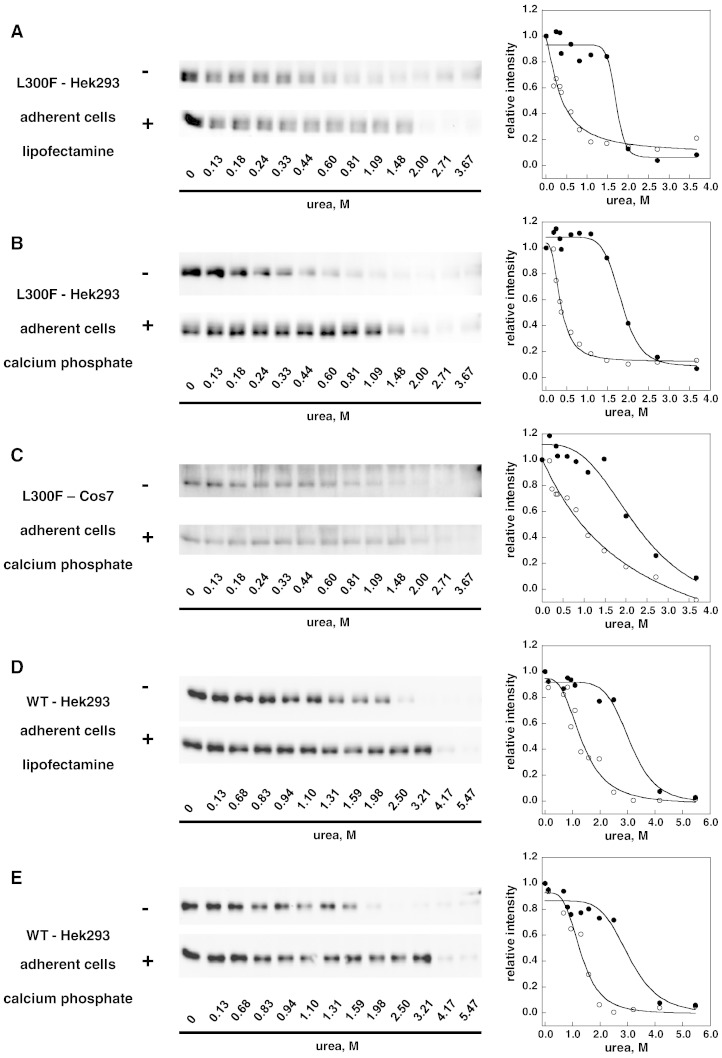
Stability enhancement of lysosomal alpha galactosidase by 1-deoxy-galactonojirimycin. Comparison between different cell hosts and transfection methods. Lysates of COS7 or HEK293 cells transfected with different methods and expressing L300F (panels A, B, and C) or wild type (panels D and E) lysosomal alpha-galactosidase were mixed with the denaturant to obtain final urea concentrations ranging from 0 to 6 M. The experiment was conducted in McIlvaine buffer at pH 7.4 either in the absence or in the presence of DGJ 40 μM. Pulse proteolysis (1 min at 37 °C, 1:5 thermolysin to substrate ratio) was performed after the equilibrium was reached to digest unfolded protein and then analyzed by western-blot. The intensity of the bands was quantified and data were expressed as fraction of the zero urea sample.

We compared the effect of different transfection protocols on the results obtained either assaying the enzymatic activity in cells exposed or not exposed to DGJ or with the novel method based on chemically induced unfolding. The experiments were carried out on L300F-AGAL. Recipient hosts were COS7 cells or HEK293, either adherent cells or in suspension, and transfection reagents were Lipofectamine® ltx, Fugene ®HD or calcium phosphate. To test the currently employed method, after transfection, the cells were supplemented or not with 0.02 mM DGJ and AGAL activity assayed in cell free extracts. The value obtained was either divided by the protein concentration (Fig. 8 panel A) or normalized to luciferase activity (Fig. 8 panel B). Responsiveness can be defined as AGAL activity of a given mutant after administration of DGJ to the cells, divided by the activity of wild type AGAL expressed in the same cells non-exposed to the drug, multiplied by 100, i.e. resp = (mut_activity + DGJ / WT_activity − DGJ × 100). Different values are obtained if the activity is normalized considering protein concentration (resp_a) or transfection efficiency (resp_b). In terms of responsiveness, the results shown in Fig. 8 are consistent although outliers are observed. We tested L300F-AGAL produced in HEK293 using lipofectamine (resp_a = 32%; resp_b = 55%), or calcium phosphate (resp_a = 68%; resp_b = 81%), or in COS7 using calcium phosphate (resp_a = 290%; resp_b = 51%) and WT-AGAL produced in HEK293 using lipofectamine or calcium phosphate with the novel method based on chemically induced unfolding. In this case the transfection was carried out on adherent cells without addition of DGJ before the lysis. Results in Fig. 9 show that the concentration of urea needed for half denaturation is little influenced by the transfection protocol.

## 3 Discussion

Pharmacological chaperones work because they stabilize mutant proteins and the method based on thermodynamic analysis is designed to assess this property straightforwardly.

Thermodynamic analysis allows the association of a quantifiable parameter to the stability of proteins in a given state. Melting temperatures, free energies or enthalpies of unfolding, midpoint denaturant concentration, and m-values help us evaluate the stability of wild type and mutant forms of a protein under different environmental conditions, pH or ligands. In particular the thermal shift assay proved to be a useful method to test pharmacological chaperones on purified proteins [35]. This method, however, cannot be exploited to analyze mutants, unless they are expressed in large amounts and purified. Such a pre-requisite is unfeasible in the majority of cases. On the other hand, urea induced denaturation can be monitored by limited proteolysis followed by electrophoresis separation exploiting the fact that unfolded proteins are more sensitive to proteases than their folded counterparts. The method can be adapted to raw extracts. We demonstrated that results obtained by limited proteolysis are consistent with those obtained by more conventional optical techniques in the case of Fabrazyme®.

Urea-induced unfolding allows the assignment of a useful measure of stability to a protein in a given state that is the half-denaturation urea concentration, C_0.5_. We demonstrated that urea-induced unfolding can be exploited to test the stability of wild type or mutant AGAL in cell extracts and the capacity of chaperones to stabilize the enzyme.

Usually the efficacy of PC is evaluated on the patient's cells or in cells transfected with an expression vector carrying the mutation and cultured in a medium supplemented or not with the drug. We compared the data obtained in different labs with this method. We found 15 responsive mutations which have been tested in different labs [19], [20], [22], [24].

We obtained the responsiveness in percentage defined as the AGAL activity of a given mutant after administration of DGJ to the cells, divided by the activity of wild type AGAL expressed in the same cells non-exposed to the drug, multiplied by 100, i.e. resp = (mut_activity + DGJ / WT_activity − DGJ × 100). We calculated an average responsiveness in percentage (resp), a standard deviation (SDresp) and an average standard deviation. The results are in qualitative accordance, but quantitatively the extent of responsiveness varies, with an average standard deviation, ∑ _*i* = 1_^15^(SDresp_i_), as large as 15 ± 10.

The approach based on chemically induced unfolding gives results that are little influenced by the method chosen to express the protein. The results are consistent with those obtained measuring the enzymatic activity in cells exposed or not exposed to the drug [27] (Fig. 7). The method based on chemically induced unfolding offers some advantages. For example it consents to test proteins at different pHs. This is important considering that, in general, chaperones are inhibitors of the enzymatic activity. The ideal drug for lysosomal storage disorders such as Fabry disease, should bind the protein in the neutral compartment where it folds and dissociates in the acidic compartment where AGAL exerts its function. Moreover the method based on chemically induced unfolding can be used to test lead compounds on mutants expressed in tiny amounts and unpurified. In this case the molecules might be yet unable to cross the cell membrane and administration in the cell medium would be not effective.

Diverse methods to test the efficacy of pharmacological chaperones on individual mutations of a given disease are urgently needed. In particular this is true for Fabry disease, a pathology characterized by hundreds of different mutations each affecting a scarce and dispersed population. Clinical trials with control groups are difficult as exemplified by studies carried out to test the efficacy of DGJ on male [12], [26] or female [36] populations. Twenty-one out of 450 missense/nonsense mutations have been tested and only in few cases was it possible to enroll more than one patient. Independent positive results obtained in vitro with the thermodynamic approach and other methods [37] might be the only support to choose the best therapy, PC or enzyme replacement therapy for individual Fabry patients.

## 4 Materials and methods

### 4.1 Cell cultures

The clone SC319065, which contains the full length cDNA for wild type human AGAL inserted into the expression vector pCMV6-AC, was purchased from Origene (Rockville, MD, USA). Q280K-AGAL, D244H-AGAL, L300F-AGAL and R301P-AGAL were obtained by site directed mutagenesis in the same vector [27]. Human embryo kidney 293 (HEK293) and African green monkey kidney (COS7) cells were maintained in Dulbecco's Modified Eagle's Medium (GIBCO BRL) with 10% fetal bovine serum, 100 units/ml penicillin, and 100 mg/ml streptomycin at 37 °C in a humidified atmosphere containing 5% CO2.

COS7 cells were transfected with individual plasmids using the Lipofectamine®2000 (Invitrogen Molecular Probes, lifetechnologies.com) cationic lipid reagent as previously described [27]. Transfected cells were plated onto a 100 mm dish and cultured in DMEM containing 10% FBS in the presence of 0.02 mM DGJ at 37 °C and 5% CO2. After a 48 h incubation, the cells were washed in PBS (5 times), scraped and harvested by centrifugation. Dry pellets were resuspended in water and lysed by freeze-thawing.

The effect of transfection method on the quantitative assessment of responsiveness to DGJ was tested. In assays carried out on adherent cells, COS7 or HEK293 was seeded into 6-well plates the day before transfection, grown to 80–90% confluency, and transfected by Lipofectamine® ltx (Invitrogen, lifetechnologies.com), Fugene® HD (Promega, Italy) or CalPhos™ Mammalian Transfection Kit (Takara Bio Europe/Clontech) according to manufacturer's with pCMV6-AC plasmids encoding either L300F-AGAL or WT-AGAL.

In assays carried out on cells in suspension, HEK293 was harvested by trypsin treatment, resuspended in DMEM containing 10% FBS and supplemented with the appropriate transfection mix solution. The cells were then distributed into wells of a six-well plate at 60% confluency and allowed to adhere. The medium was substituted by fresh DMEM, 10% FBS (3 ml).

Cells were cultivated with or without 0.02 mM DGJ for 48 h, rinsed 5 times in PBS, scraped and harvested by centrifugation. Dry pellets were resuspended in 50 μl of water and lysed by freeze-thawing The efficiency of the transfection was calculated by cotransfecting a 1:4 ratio of pMIR vector (Applied Biosystems/Ambion, Italy) containing the luciferase gene and assaying the reporter gene activity under standard conditions using ONE-Glo™ Luciferase Assay System (Promega, Italy). AGAL activity was measured by adding 2 μl of the cell lysates to 38 μl of AGAL assay buffer (sodium citrate 27 mM-sodium phosphate dibasic 46 mM, 4-methylumbelliferyl-alpha-d-galactopyranoside 5 mM and N-acetyl-d-galactosamine 100 mM, pH 4.5) and incubated for 1 h at 37 °C. All chemicals were obtained from Sigma (SIGMA, Milan, Italy). The reaction was stopped by adding 360 μl of 1 M sodium carbonate buffer [20]. Fluorescence was detected using a fluorescence spectrophotometer (Cary Eclypse-Varian) at 355 nm excitation and 460 nm emission. A 4-methylumbelliferone standard curve ranging from 5 nM to 25 μM was run in parallel for conversion of fluorescence data to AGAL activity expressed as nmol/mg protein per hour.

### 4.2 Urea-induced unfolding

Fabrazyme® (Genzyme, Cambridge, MA) (0.3 mg/ml) was induced to unfold by urea in McIlvaine buffer at pH 7.4, with or without 0.04 mM DGJ in a final volume of 0.150 ml. Dilutions of the enzyme reconstituted in water, buffer and 8 M urea were carried out in separate tubes in order to obtain the desired final conditions where urea concentration varied from 0 to 6 M. Molar ellipticity per mean residue [ϑ]_MRE@223_ in deg cm^2^ dmol^− 1^ was calculated from the equation [ϑ]_MRE@223_ = 100∙[ϑ]_obs_*l*∙ *l* C where [ϑ]_obs_ is the ellipticity measured in degrees on a JASCO J-815 spectropolarimeter, *l* is the pathlength of the cell in cm and C is the protein concentration referred to the mean residue molecular weight. Aliquots were withdrawn from the same samples prepared for CD measures, supplemented with CaCl_2_ (10 mM final concentration) and with the appropriate amount of thermolysin necessary to realize a 1:5 protease to protein ratio. After 1 min incubation at 37 °C the reaction was stopped by addition of EDTA (40 mM final concentration). Folded undigested proteins were quantified with a ChemiDoc XRS (Bio-Rad Laboratories, Hercules, CA-USA) systems after having been separated by SDS-PAGE and colored by Coomassie Blue Staining.

Urea-induced unfolding of COS7 or HEK293 cell lysates containing wild-type AGAL or L300F-AGAL, or Q280K-AGAL, or D244H-AGAL, or R301P-AGAL was conducted in McIlvaine buffer at pH 4.5, 5.2 or 7.4, in a total volume of 17 μl containing 0.2 mg/ml protein, urea ranging from 0 to 6 M, in the presence or in the absence of 0.04 mM DGJ. The samples were incubated for 16–18 h at 20 °C.

Pulse proteolysis was performed after the equilibrium was reached to digest unfolded proteins. The samples at pH 5.2 or 7.4 were treated with appropriate amount of thermolysin in order to realize a 1:5 protease to substrate (total proteins) ratio. CaCl_2_ was also added to the reaction mixture up to 10 mM. After 1 min incubation at 37 °C the reaction was stopped by addition of EDTA (40 mM final concentration). The samples at pH 4.2 were treated with the appropriate amount of pepsin in order to realize a 1:5 protease to substrate (total proteins) ratio. After 1 min incubation at 37 °C the protease was inactivated by addition of Tris 1 M pH 9.0.

Aliquots (1 μg) were loaded onto a 12% SDS-PAGE gel and transferred to PVDF membrane (Bio-Rad Laboratories, Hercules, CA-USA) for 2 h at 100 V in Tris-Glycine buffer containing 20% methanol. The membrane was blocked with 5% (w/v) non-fat dried skimmed milk in TBS containing Tween20 0.05% (TTBS) at 4 °C overnight, and then treated with the primary antibody (polyclonal antibody produced in rabbit, Abcam 70520) diluted in TTBS 1:500 for 1 h at room temperature. After washing with an excess of TTBS, the membrane was treated with the secondary antibody (HRP-conjugated anti-rabbit IgG antibody produced in goat, Bio-Rad 1706515) diluted in the TTBS solution 1:2000 for 1 h at room temperature. After washing, the detection was performed by using the Immun-Star WesternC chemiluminescence detection kit (Bio-Rad Laboratories, Hercules, CA-USA).

### 4.3 Miscellaneous

N-Glycosidase F was purchased by Roche (Roche Diagnostics GmbH, Mannheim, Germany). Deglycosylation of Fabrazyme® or AGAL expressed in COS7 cells (9 μg, 0.3 mg/ml) was performed according to the producer's instructions. Briefly, EDTA and SDS were added to the proteins (final concentrations were 20 mM and 0.1% respectively). The samples were boiled for 5 min, immediately cooled before the addition of NP-40 to a final condition of 0.7% and N-Glycosidase F (1 unit) and incubated overnight at 37 °C. Controls were run in parallel without the addition of N-Glycosidase F. Aliquots were analyzed by SDS-PAGE and western blotting.

SDS-PAGE was performed using standard procedures [38].

Protein concentrations were routinely estimated using the Bio-Rad Protein System, with bovine serum albumin as the standard. Protease concentrations were determined by using the appropriate extinction coefficient (E1%/280 = 17.65 thermolysin, E1%/280 = 14.7 pepsin).

Graph plotting and curve fitting were carried out with KaleidaGraph (Synergy Software, PA).

## References

[1] Yue P., Li Z., Moult J. (2005). Loss of protein structure stability as a major causative factor in monogenic disease. J. Mol. Biol..

[2] Boyd R.E., Lee G., Rybczynski P., Benjamin E.R., Khanna R., Wustman B.A., Valenzano K.J. (2013). Pharmacological chaperones as therapeutics for lysosomal storage diseases. J. Med. Chem..

[3] Ishii S. (2012). Pharmacological chaperone therapy for Fabry disease. Proc. Jpn. Acad. Ser. B Phys. Biol. Sci..

[4] Young-Gqamana B., Brignol N., Chang H.H., Khanna R., Soska R., Fuller M., Sitaraman S.A., Germain D.P., Giugliani R., Hughes D.A., Mehta A., Nicholls K., Boudes P., Lockhart D.J., Valenzano K.J., Benjamin E.R. (2013). Migalastat HCl reduces globotriaosylsphingosine (lyso-Gb3) in Fabry transgenic mice and in the plasma of Fabry patients. PLoS One.

[5] Benito J.M., Garcia Fernandez J.M., Ortiz Mellet C. (2011). Pharmacological chaperone therapy for Gaucher disease: a patent review. Expert. Opin. Ther. Pat..

[6] Khanna R., Benjamin E.R., Pellegrino L., Schilling A., Rigat B.A., Soska R., Nafar H., Ranes B.E., Feng J., Lun Y., Powe A.C., Palling D.J., Wustman B.A., Schiffmann R., Mahuran D.J., Lockhart D.J., Valenzano K.J. (2010). The pharmacological chaperone isofagomine increases the activity of the Gaucher disease L444P mutant form of beta-glucosidase. FEBS J..

[7] Flanagan J.J., Rossi B., Tang K., Wu X., Mascioli K., Donaudy F., Tuzzi M.R., Fontana F., Cubellis M.V., Porto C., Benjamin E., Lockhart D.J., Valenzano K.J., Andria G., Parenti G., Do H.V. (2009). The pharmacological chaperone 1-deoxynojirimycin increases the activity and lysosomal trafficking of multiple mutant forms of acid alpha-glucosidase. Hum. Mutat..

[8] Khanna R., Flanagan J.J., Feng J., Soska R., Frascella M., Pellegrino L.J., Lun Y., Guillen D., Lockhart D.J., Valenzano K.J. (2012). The pharmacological chaperone AT2220 increases recombinant human acid alpha-glucosidase uptake and glycogen reduction in a mouse model of Pompe disease. PLoS One.

[9] Santos-Sierra S., Kirchmair J., Perna A.M., Reiss D., Kemter K., Roschinger W., Glossmann H., Gersting S.W., Muntau A.C., Wolber G., Lagler F.B. (2012). Novel pharmacological chaperones that correct phenylketonuria in mice. Hum. Mol. Genet..

[10] Germain D.P. (2010). Fabry disease. Orphanet J. Rare Dis..

[11] Deegan P.B. (2012). Fabry disease, enzyme replacement therapy and the significance of antibody responses. J. Inherit. Metab. Dis..

[12] Germain D.P., Giugliani R., Hughes D.A., Mehta A., Nicholls K., Barisoni L., Jennette C.J., Bragat A., Castelli J., Sitaraman S., Lockhart D.J., Boudes P.F. (2012). Safety and pharmacodynamic effects of a pharmacological chaperone on alpha-galactosidase A activity and globotriaosylceramide clearance in Fabry disease: report from two phase 2 clinical studies. Orphanet J. Rare Dis..

[13] Andreotti G., Guarracino M.R., Cammisa M., Correra A., Cubellis M.V. (2010). Prediction of the responsiveness to pharmacological chaperones: lysosomal human alpha-galactosidase, a case of study. Orphanet J. Rare Dis..

[14] Siekierska A., De Baets G., Reumers J., Gallardo R., Rudyak S., Broersen K., Couceiro J., Van Durme J., Schymkowitz J., Rousseau F. (2012). alpha-Galactosidase aggregation is a determinant of pharmacological chaperone efficacy on Fabry disease mutants. J. Biol. Chem..

[15] Cammisa M., Correra A., Andreotti G., Cubellis M.V. (2013). Fabry_CEP: a tool to identify Fabry mutations responsive to pharmacological chaperones. Orphanet J. Rare Dis..

[16] Cammisa M., Correra A., Andreotti G., Cubellis M.V. (2013). Identification and analysis of conserved pockets on protein surfaces. BMC Bioinforma..

[17] Yam G.H., Bosshard N., Zuber C., Steinmann B., Roth J. (2006). Pharmacological chaperone corrects lysosomal storage in Fabry disease caused by trafficking-incompetent variants. Am. J. Physiol. Cell Physiol..

[18] Ishii S., Chang H.H., Kawasaki K., Yasuda K., Wu H.L., Garman S.C., Fan J.Q. (2007). Mutant alpha-galactosidase A enzymes identified in Fabry disease patients with residual enzyme activity: biochemical characterization and restoration of normal intracellular processing by 1-deoxygalactonojirimycin. Biochem. J..

[19] Shin S.H., Kluepfel-Stahl S., Cooney A.M., Kaneski C.R., Quirk J.M., Schiffmann R., Brady R.O., Murray G.J. (2008). Prediction of response of mutated alpha-galactosidase A to a pharmacological chaperone. Pharmacogenet. Genomics.

[20] Benjamin E.R., Flanagan J.J., Schilling A., Chang H.H., Agarwal L., Katz E., Wu X., Pine C., Wustman B., Desnick R.J., Lockhart D.J., Valenzano K.J. (2009). The pharmacological chaperone 1-deoxygalactonojirimycin increases alpha-galactosidase A levels in Fabry patient cell lines. J. Inherit. Metab. Dis..

[21] Filoni C., Caciotti A., Carraresi L., Cavicchi C., Parini R., Antuzzi D., Zampetti A., Feriozzi S., Poisetti P., Garman S.C., Guerrini R., Zammarchi E., Donati M.A., Morrone A. (2010). Functional studies of new GLA gene mutations leading to conformational Fabry disease. Biochim. Biophys. Acta.

[22] Fan J.Q., Ishii S. (2007). Active-site-specific chaperone therapy for Fabry disease. Yin and Yang of enzyme inhibitors. FEBS J..

[23] Spada M., Pagliardini S., Yasuda M., Tukel T., Thiagarajan G., Sakuraba H., Ponzone A., Desnick R.J. (2006). High incidence of later-onset Fabry disease revealed by newborn screening. Am. J. Hum. Genet..

[24] Shimotori M., Maruyama H., Nakamura G., Suyama T., Sakamoto F., Itoh M., Miyabayashi S., Ohnishi T., Sakai N., Wataya-Kaneda M., Kubota M., Takahashi T., Mori T., Tamura K., Kageyama S., Shio N., Maeba T., Yahagi H., Tanaka M., Oka M., Sugiyama H., Sugawara T., Mori N., Tsukamoto H., Tamagaki K., Tanda S., Suzuki Y., Shinonaga C., Miyazaki J., Ishii S., Gejyo F. (2008). Novel mutations of the GLA gene in Japanese patients with Fabry disease and their functional characterization by active site specific chaperone. Hum. Mutat..

[25] Kim M.S., Song J., Park C. (2009). Determining protein stability in cell lysates by pulse proteolysis and Western blotting. Protein Sci..

[26] Ferri L., Guido C., la Marca G., Malvagia S., Cavicchi C., Fiumara A., Barone R., Parini R., Antuzzi D., Feliciani C., Zampetti A., Manna R., Giglio S., Della Valle C.M., Wu X., Valenzano K.J., Benjamin R., Donati M.A., Guerrini R., Genuardi M., Morrone A. (2011). Fabry disease: polymorphic haplotypes and a novel missense mutation in the GLA gene. Clin. Genet..

[27] Andreotti G., Citro V., De Crescenzo A., Orlando P., Cammisa M., Correra A., Cubellis M.V. (2011). Therapy of Fabry disease with pharmacological chaperones: from in silico predictions to in vitro tests. Orphanet J. Rare Dis..

[28] Lieberman R.L., Aquino A.D., Ringe J.D., Petsko G.A. (2009). Effects of pH and iminosugar pharmacological chaperones on lysosomal glycosidase structure and stability. Biochemistry.

[29] Pace C.N., Marshall H.F. (1980). A comparison of the effectiveness of protein denaturants for beta-lactoglobulin and ribonuclease. Arch. Biochem. Biophys..

[30] Guce A.I., Clark N.E., Rogich J.J., Garman S.C. (2011). The molecular basis of pharmacological chaperoning in human alpha-galactosidase. Chem. Biol..

[31] Shabbeer J., Robinson M., Desnick R.J. (2005). Detection of alpha-galactosidase a mutations causing Fabry disease by denaturing high performance liquid chromatography. Hum. Mutat..

[32] Dobrovolny R., Dvorakova L., Ledvinova J., Magage S., Bultas J., Lubanda J.C., Elleder M., Karetova D., Pavlikova M., Hrebicek M. (2005). Relationship between X-inactivation and clinical involvement in Fabry heterozygotes. Eleven novel mutations in the alpha-galactosidase A gene in the Czech and Slovak population. J. Mol. Med. (Berl).

[33] Topaloglu A.K., Ashley G.A., Tong B., Shabbeer J., Astrin K.H., Eng C.M., Desnick R.J. (1999). Twenty novel mutations in the alpha-galactosidase A gene causing Fabry disease. Mol. Med..

[34] Ashley G.A., Shabbeer J., Yasuda M., Eng C.M., Desnick R.J. (2001). Fabry disease: twenty novel alpha-galactosidase A mutations causing the classical phenotype. J. Hum. Genet..

[35] Benjamin E.R., Khanna R., Schilling A., Flanagan J.J., Pellegrino L.J., Brignol N., Lun Y., Guillen D., Ranes B.E., Frascella M., Soska R., Feng J., Dungan L., Young B., Lockhart D.J., Valenzano K.J. (2012). Co-administration with the pharmacological chaperone AT1001 increases recombinant human alpha-galactosidase A tissue uptake and improves substrate reduction in Fabry mice. Mol. Ther..

[36] Giugliani R., Waldek S., Germain D.P., Nicholls K., Bichet D.G., Simosky J.K., Bragat A.C., Castelli J.P., Benjamin E.R., Boudes P.F. (2013). A Phase 2 study of migalastat hydrochloride in females with Fabry disease: selection of population, safety and pharmacodynamic effects. Mol. Genet. Metab..

[37] Wu X., Katz E., Della Valle M.C., Mascioli K., Flanagan J.J., Castelli J.P., Schiffmann R., Boudes P., Lockhart D.J., Valenzano K.J., Benjamin E.R. (2011). A pharmacogenetic approach to identify mutant forms of alpha-galactosidase A that respond to a pharmacological chaperone for Fabry disease. Hum. Mutat..

[38] Laemmli U.K. (1970). Cleavage of structural proteins during the assembly of the head of bacteriophage T4. Nature.

